# Application of immersive virtual reality in the training of wheelchair boxers: evaluation of exercise intensity and users experience additional load– a pilot exploratory study

**DOI:** 10.1186/s13102-024-00878-6

**Published:** 2024-04-10

**Authors:** Jacek Polechoński, Alan Langer, Anna Akbaş, Anna Zwierzchowska

**Affiliations:** 1 Institute of Sport Sciences, Academy of Physical Education in Katowice, Katowice, Poland; 2 Student Scientific Circle of Physical Activity and Tourism in Virtual Reality “ACTIVE VR”, Academy of Physical Education in Katowice, Katowice, Poland

**Keywords:** Wheelchair boxing, Immersive virtual reality, Physical activity, Paraboxing, Disabled athletes

## Abstract

**Background:**

Over the last few years, there has been a growing interest in workout apps and active virtual reality video games (AVRGs), which provide entertainment and enable users to undertake various forms of physical activity (PA) at home. Presumably, these types of exercises can be particularly useful for people with physical disabilities, who experience problems with access to sports and leisure facilities due to architectural and communication barriers. However, it is interesting whether the intensity of PA in VR is high enough to provide users with health benefits, as it is mainly based on arm movements.

**Objective:**

The main aim of the study was to evaluate the intensity of physical exercise of wheelchair boxers during a boxing training session using the FitXR app in immersive VR in light of health-related PA recommendations. The effect of Velcro-fastened hand-held weights (HHWs) on the intensity of PA undertaken by people in VR was also examined, and the attractiveness of virtual exercise were assessed in the opinion of users.

**Methods:**

PA intensity was evaluated using a heart rate monitor based on the percentage of maximal heart rate (% HRmax) and the Borg’s rating of perceived exertion (RPE 6–20). The attractiveness perceived during exercise by users were evaluated using the Physical Activity Enjoyment Scale (PACES 1–7 scale).

**Results:**

The study shows that the exercise intensity of the athletes during wheelchair boxing training in VR is at a beneficial moderate level for health (HR_ave_=68.98% HR_max_). The use of HHWs (0.5 kg) does not significantly increase the PA intensity of the individuals during virtual exercise. Users with disabilities highly rated the attractiveness (6.32 ± 0.79 points) of PA during virtual boxing training.

**Conclusions:**

Boxing exercises in VR can be an attractive and health-related form of PA for wheelchair boxers and a supplement to their conventional training.

**Supplementary Information:**

The online version contains supplementary material available at 10.1186/s13102-024-00878-6.

## Background

Immersive virtual reality (VR) is an artificial reality in which a person is completely immersed in a computer-generated world. This is possible, for example, by using a head-mounted display (HMD) and displaying three-dimensional images right in front of the user’s eyes [1]. In this way, the user is cut off from the visual and auditory stimuli of the real environment and instead receives images, sound, and even tactile sensations of the simulated world. Over the past few years, there has been a dynamic development of VR and growing interest in workout apps and active virtual reality video games (AVRGs), which provide entertainment and enable users to undertake various forms of physical activity (PA). There have been studies on the intensity of PA in VR [2–5], analysis of body movements in an immersive virtual environment [6, 7], the evaluation of the enjoyment of this type of physical exercise [3, 4, 8–12], and the possibility of diagnosing and shaping motor and cognitive abilities in a virtual world [13–16].

The VR is increasingly being used to improve the function of people with various dysfunctions. VR technology may provide benefits in the rehabilitation of individuals with Parkinson’s disease, multiple sclerosis, anxiety, heart disorders, cancer, spinal cord injury, those recovering from ACL reconstruction and older adults [17–25]. The collective findings from those studies demonstrate reduction in stress level and influence of emotional well-being, improve balance, gait and upper limb function and improvement in cognitive skills. The use of VR in the rehabilitation process is also supported by the fact that patients who perform exercises in a virtual environment report less pain [26, 27]. The pain-reducing effect of the immersive virtual environment is so significant that VR can be an effective therapeutic support in burn wound care [28] and during exercises to increase the range of motion in burned joints [29]. The specific properties of immersive virtual reality are also used to alleviate pain when undertaking various medical procedures in children and pediatric rehabilitation [30] and even in women during childbirth [31].

As seen from the literature review, the spectrum of applications of VR in rehabilitation is vast. However, there is a lack of research on the use of this technology in rehabilitation and sports training for wheelchair users. The few papers focus on simulating wheelchair use in a virtual environment [32–35]. Little is known about the sensations of people with disabilities using VR technology to practice PA, their opinions on the usefulness of such solutions, and the intensity of this type of physical activity. In addition to the therapeutic aspect, applications using immersive VR can be useful for wheelchair users who participate in recreational or competitive sports. Such people, due to architectural and communication barriers, have difficult access to sports and leisure facilities, and thanks to VR, they can exercise at home in a small space. It is worth emphasizing that thanks to existing interactive training programs, it is possible to practice various forms of PA in a virtual environment. The great potential of immersive VR technology in sports is evidenced by the fact that it is increasingly being used by trainers in the process of improving physical fitness [13, 14, 36–38] and even teaching movement skills. There is already evidence that skills acquired in VR can be transferred to the real world [39–41].

One of the newly developed forms of physical activity for people with physical disabilities is wheelchair boxing (paraboxing). Paraboxing is practiced by people with various musculoskeletal dysfunctions. The most common paraboxers are amputees, people with cerebral palsy, and tetra- and paraplegics. Some of them practice the sport recreationally. Their training is based on performing punches on punching shields, bags, and other equipment, and practicing dodging. Some take up boxing against a real opponent. The most common fighting format is boxing bouts, in which the fighters sit close together (arm’s reach distance) on immobilized wheelchairs. This type of fight is quite brutal, as the fighters take a fair number of blows, most of which are received to the head. To avoid them, they can only balance their bodies, which is rather limited due to their seated position, while they cannot move away from their opponent, as in traditional boxing. There is also a fighting formula where competitors can move around in wheelchairs. The need to take a large number of punches in paraboxing makes competition dangerous for athletes, especially those with dysfunctions located in the spine. This article ignores the aspect of the advisability of paraboxing as a sport. It is not the intention of the authors to decide whether disabled people should engage in extreme and risky forms of PA, especially as there are many other dangerous sports besides boxing that are preferred by disabled athletes (e.g. downhill skiing or water skiing). Like able-bodied athletes, people with disabilities are looking for an adrenaline rush, disregarding the risk of injury or accident, and they ultimately decide what form of PA they want to practice.

The risks associated with undertaking wheelchair training can be significantly minimized by using immersive VR technology, which allows exercisers to simultaneously experience sensations and stimuli similar to those experienced during real exercise [33, 42, 43]. The application used in our study enables realistic training involving the delivery of various boxing punches and performing blocks, which can foster the development of motor skills useful to boxers. Effective training should also be characterized by sufficient intensity to improve the athletes’ physical capacity. It is interesting whether this type of PA is intensive enough to guarantee that users are provided with health benefits. It should be noted that PA when performing wheelchair boxing techniques relies only on arm movements and small movements of the torso, which may not guarantee adequate exercise intensity.

Therefore, the main aim of this pilot exploratory study was to evaluate PA intensity during boxing training in VR using the FitXR app in the context of health recommendations for PA. We aimed to assess what is the exercise intensity level of paraboxers during boxing training using the FitXR app and if it is sufficient to be considered a health-promoting PA. Since PA based mainly on arm movements may not be intensive enough to meet these recommendations, we also assessed the effect of additional loading in the form of hand-held weights on the exercise load in disabled athletes. Furthermore, an attempt was made to assess whether this form of PA is attractive to paraboxers and whether they find it a useful training tool. We hypothesize that the paraboxers engaged in VR boxing training will demonstrate exercise intensity levels within the recommended range for health promotion. Additionally, we hypothesize that the incorporation of hand-held weights will significantly increase exercise intensity. Finally, we anticipate that paraboxers will find this virtual training modality attractive and consider it a valuable tool for their training regimen.

## Methods

The research was carried out at the Sports Centre for Persons with Disabilities in the Hala 100-lecia sports hall in the KS Cracovia club in Krakow, Poland, according to the procedures of the certified Laboratory of Research on Pro-Health Physical Activity (PN-EN ISO 9001:2015, certificate validity: 7.12.2021–16.12.2024). The study involved 11 male wheelchair boxers working with the Warriors Foundation (Polish: Fundacja Wojownicy) (age: 30.0 ± 7.3 years, body height: 170.6 ± 14.0 cm; body weight: 68.5 ± 17.1 kg). The athletes declared a training volume of 3 to 5 h per week (4 people) and 5 to 8 h (5 people). The other two paraboxers trained more than 12 h a week. The training experience of the athletes was relatively low at 1.86 ± 1.70 years, which is because paraboxing is a newly established sport and only a few athletes in Poland have been training for more than two years. All players declared previous experience with immersive VR. The people surveyed were characterized by different types of disabilities. According to the interview, the following dysfunctions were present in the group of paraboxers: spinal cord injuries (lumbar − 4 people, thoracic − 1 person, cervical − 1 person), cerebral palsy (4 people), spina bifida in the thoracic region (1 person), and motor polyneuropathy (1 person). The study included individuals who had no problem operating the VR kit and the application used in the research. However, according to the adopted criteria, the following persons were excluded: those sensitive to flashing lights or image layouts present in programs and video games, those with symptoms of motion sickness, those suffering from epilepsy or with a history of epileptic seizures, those with physical limitations (e.g. injuries) that could hinder PA in VR, those taking agents that affect heart rate, and people who had previously used the FitXR app. The study included athletes with training experience of at least six months. It should be noted that the population of wheelchair boxers in Polish clubs and associations on the day of the study was only a few dozen. Therefore, although the group of athletes surveyed appears small, it represents a relatively large percentage of Polish paraboxers. The study was conducted according to the guidelines of the Declaration of Helsinki and reviewed and approved by the Research Ethics Committee of the Academy of Physical Education in Katowice (protocol 9/2018, annex KB/27/2022).

For VR immersion, we used the Oculus Quest 2 wireless VR headset (Facebook Technologies, LLC. 1 Hacker Way, Menlo Park, CA 94,025, USA) consisting of VR glasses and controllers. A FitXR app was used for training, including boxing training among its many exercise options. A beginner option called Fight Formation (duration of 15 min) with Rooftop (day) location/scenery. Standard settings were retained. The training was a form of active video game that involves the user delivering various boxing punches (straight, hook, undercut) at incoming targets and performing blocks when a two-glove symbol appears in front of the player. The user hits the yellow targets with the right hand, and the blue targets with the left hand. The type of punches depends on how the targets are set up, which suggests to the user which punch should be performed. During the game, the player scores points, which are calculated according to the number and accuracy of hits and the dynamics of the strike.

**Fig. 1:**
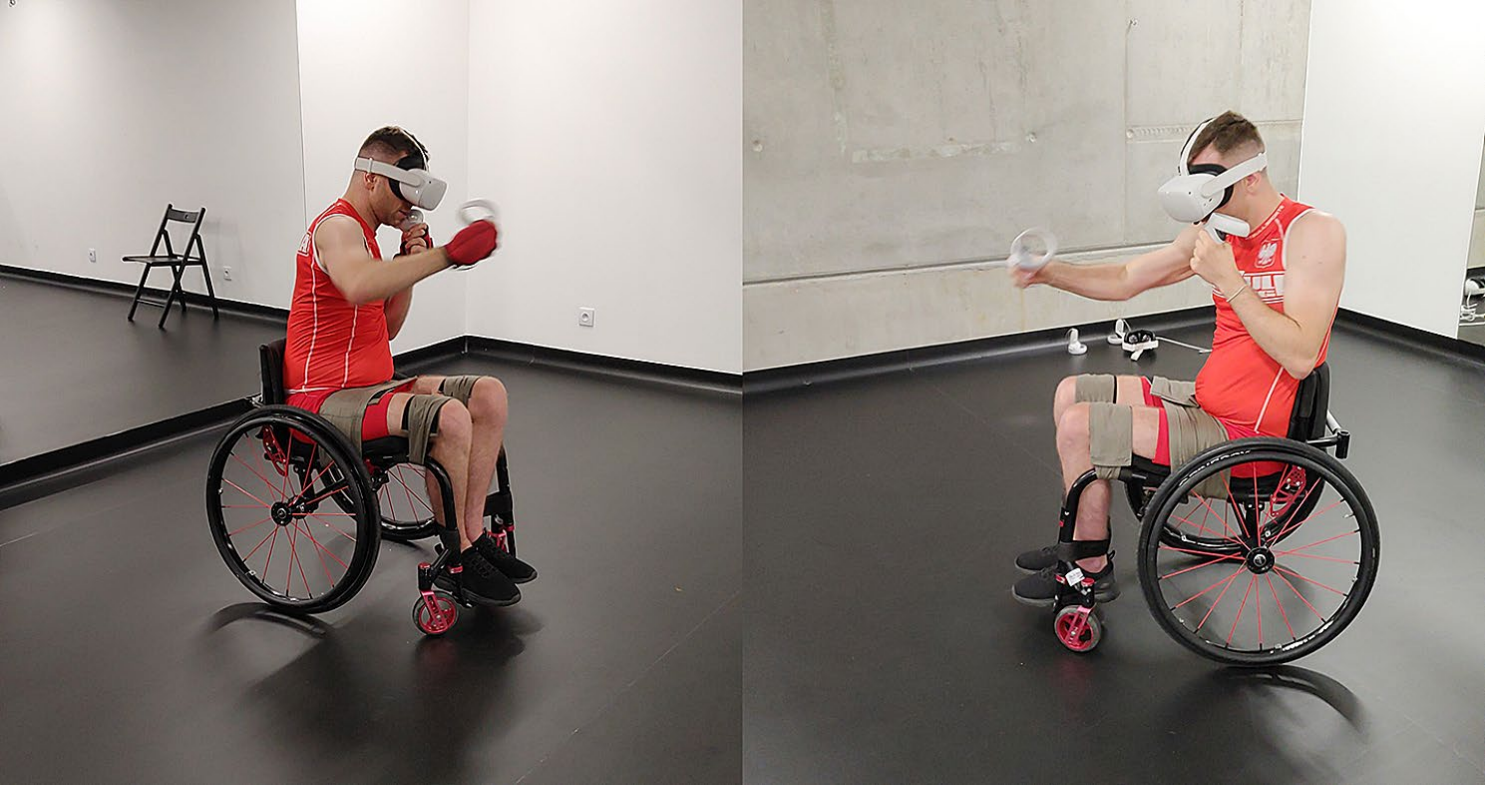
Participant during training using the FitXR app: Fight Formation workout: (**a**) with HHWs, (**b**) without HHWs

**Fig. 2:**
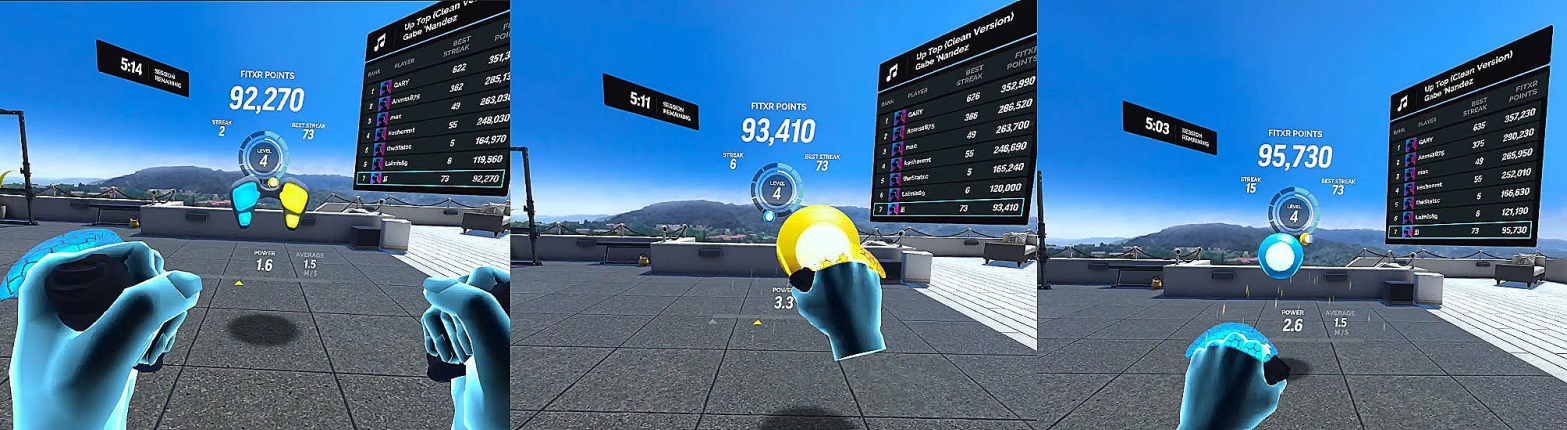
FitXR: Fight Formation workout, screenshot

Each participant was familiarized in the use of the app before participation in the study. The research procedure consisted of two training sessions. One session used an extra load to the upper limbs and the other was done without a load. Each session lasted 15 min. Subsequent subjects began alternately with sessions with and without load (crossover procedure). The sessions were separated by a 30-minute passive rest break, during which the study participants could only drink water. Two Velcro-fastened hand-held weights (HHWs), one for each hand, were used to load the upper limbs. Figure 1a and b illustrate the test stand and one of the participants, while Fig. 2 presents selected screenshots from the application used (game environment).

During training in VR, heart rates were monitored using a Vantage V heart rate monitor (Polar Electro Oy, Kempele, Finland). The level of exercise intensity was estimated from the average percentage of maximum heart rate (%HR_max_), and the average exercise heart rate (HR_ave_) was also calculated. The maximum heart rate (HR_max_) was estimated before the study from the formula 208 − 0.7× age [44]. The results obtained were compared to the PA intensity standards recommended by the American College of Sports Medicine (ACSM) [45]. According to the ACSM classification, it is assumed that during low-intensity PA, HR_ave_ <64%HR_max_. Moderate efforts are those during which HR_ave_ ≥ 64%HR_max_ and less than 77%HR_max_. High-intensity PA, on the other hand, occurs when HR_ave_ ≥77% of HR_max_. The results were also categorized into exercise intensity zones, using the Polar Flow software that works with the heart rate monitor. The average absolute duration of PA (in seconds) was estimated for the following zones: 0– less than 50% HR_max_, II– 50–59% HR_max_, III– 60–69% HR_max_, IV– 70–79% HR_max_, V– 80–89% HR_max_, and VI– ≥90% HR_max_.

In addition to objective measurements taken with the heart rate monitor, a subjective assessment of exercise intensity was also recorded. For this purpose, the Borg’s rating of perceived exertion (RPE) (6–20) was employed [46, 47]. This allowed the results of objective measurements to be correlated with the subjective perceptions of the participants. User satisfaction with PA in immersive VR practiced with and without HHWs was also assessed using questionnaire surveys. A short version of the Physical Activity Enjoyment Scale (PACES) was used [48], consisting of 8 statements to which participants responded on a 7-point Likert scale (1 = strongly disagree, 7 = strongly agree): “I find it pleasurable”, “It’s a lot of fun”, “It’s very pleasant”, “It’s very invigorating”, “It’s very gratifying”, “It’s very exhilarating”, “It’s very stimulating”, “It’s very refreshing”. An average was calculated based on the points assigned to each statement, which was the final score of the survey. The aforementioned questionnaires were completed by the participants twice: during the interval between training sessions and at the end of both sessions.

Statistical calculations were performed using Statistica v.13 (TIBCO Software Inc.) and Jamovi v. 2.2.3.0 software. Basic descriptive statistics (arithmetic means, standard deviations, and structure indices) were calculated. The normality of distribution was assessed using the Shapiro-Wilk test. The non-parametric Wilcoxon test was used to assess the significance of differences between the compared results. The level of statistical significance was set at α = 0.05. The effect size was estimated using the rank-biserial correlation coefficient (r_rb_). The Spearman’s rank correlation coefficient (r_S_) was used as a measure of the relationship between objective and subjective intensity ratings.

## Results

### PA intensity during boxing training in VR with and without HHWs in the light of health recommendations

**Fig. 3:**
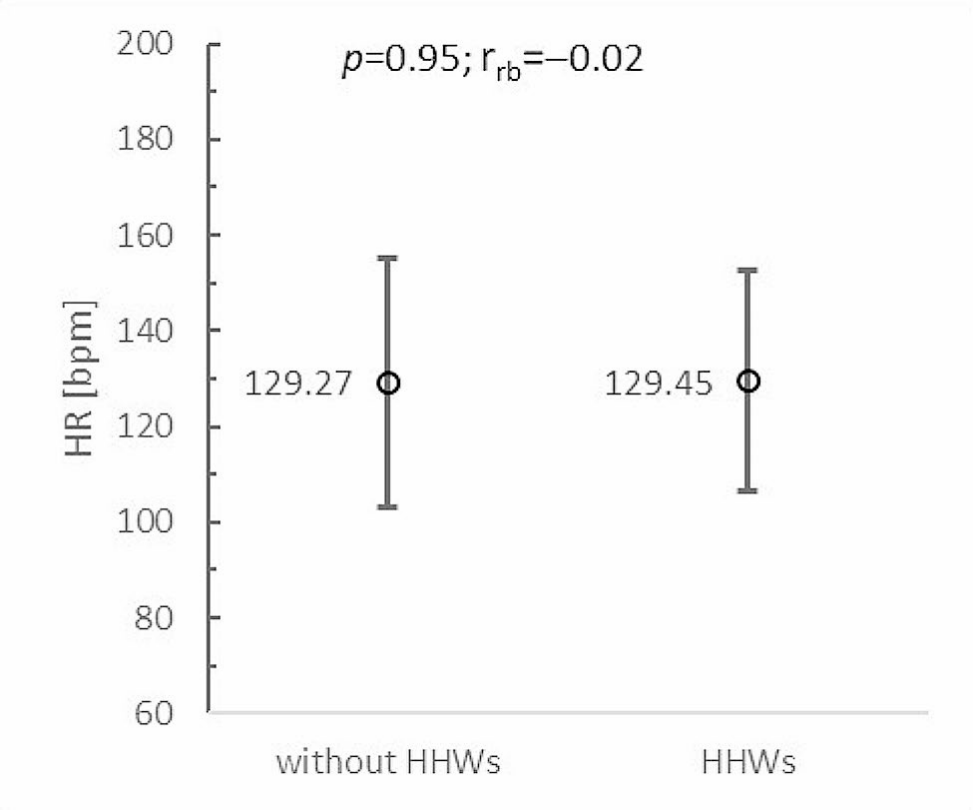
Mean heart rate of wheelchair boxers during boxing training in VR vs. upper limb load: HHWs - hand-held weights; bpm - beats per minute; *p* - *p-value*; r_rb_ - rank-biserial correlation coefficient

**Fig. 4:**
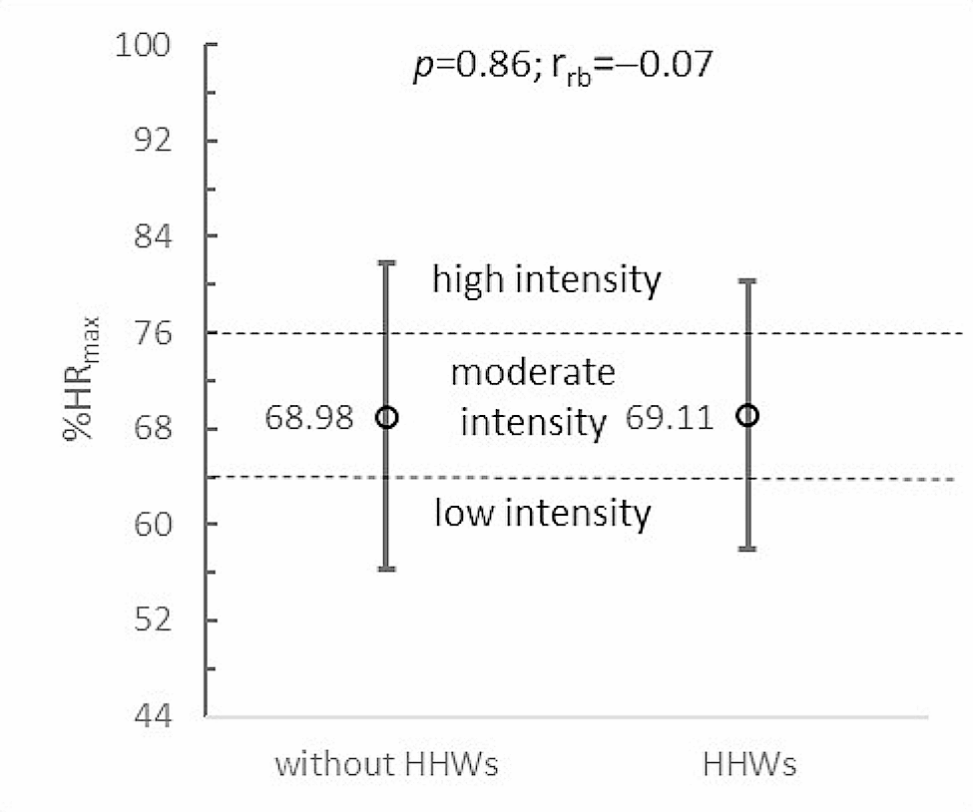
Exercise intensity of wheelchair boxers during boxing training in VR vs. upper limb loading: HHWs - hand-held weights; HR_max_ - maximum heart rate; *p* - *p*-value; r_rb_ - rank-biserial correlation coefficient

The results of the study show that additional upper limb loading in the form of HHWs did not increase the heart rate of paraboxers during boxing training in VR (*p* = 0.95; r_rb_=-0.02). Without HHWs, the mean heart rate was 129.27 ± 25.98 beats per minute (bpm), while with HHWs, it was almost identical, at 129.45 ± 22.95 bpm (Fig. 3). The results translate into the level of PA intensity, which also did not change significantly with the application of additional upper limb loading (*p* = 0.86; r_rb_=-0.07). In both training conditions, very similar average percentages of maximum heart rate (%HR_max_) were recorded in wheelchair boxers. In the case of training with HHWs, this parameter was 68.98 ± 12.77%HR_max_, while without HHWs, it was 69.11 ± 11.23%HR_max_. It should be noted that both values were in the range of 64–76%HRmax, indicating moderate exercise intensity and the beneficial nature of PA on participants’ health (Fig. 4).

**Fig. 5:**
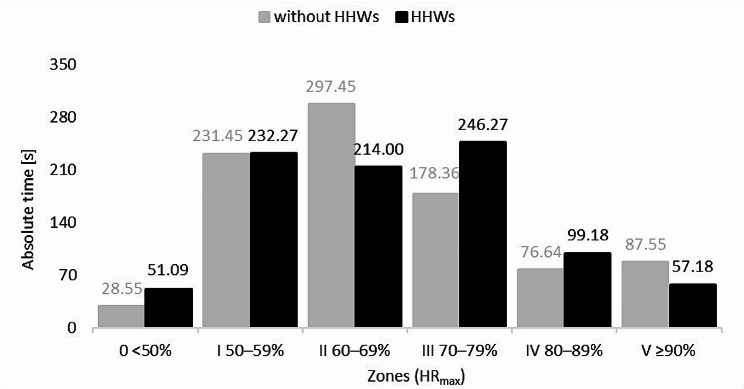
Average time spent in different heart rate zones by boxers vs. upper limb loading: HHWs - hand-held weights; HR_max_ - maximum heart rate

Analysis of both training sessions reveals that, irrespective of the upper limb load, the paraboxers’ heart rates were maintained longest in the three zones: Zone I (50–59% HR_max_), Zone II (60–69% HR_max_), and Zone III (70–79% HR_max_). The greatest differences in results were recorded in Zone II (83.45% HR_max_) and Zone III (67.91% HR_max_), but they were statistically insignificant in both the former (*p* = 0.18; r_rb_=0.46), and the latter (*p* = 0.40; r_rb_=-0.33). In the other zones, differences were minimal (Fig. 5).

**Fig. 6:**
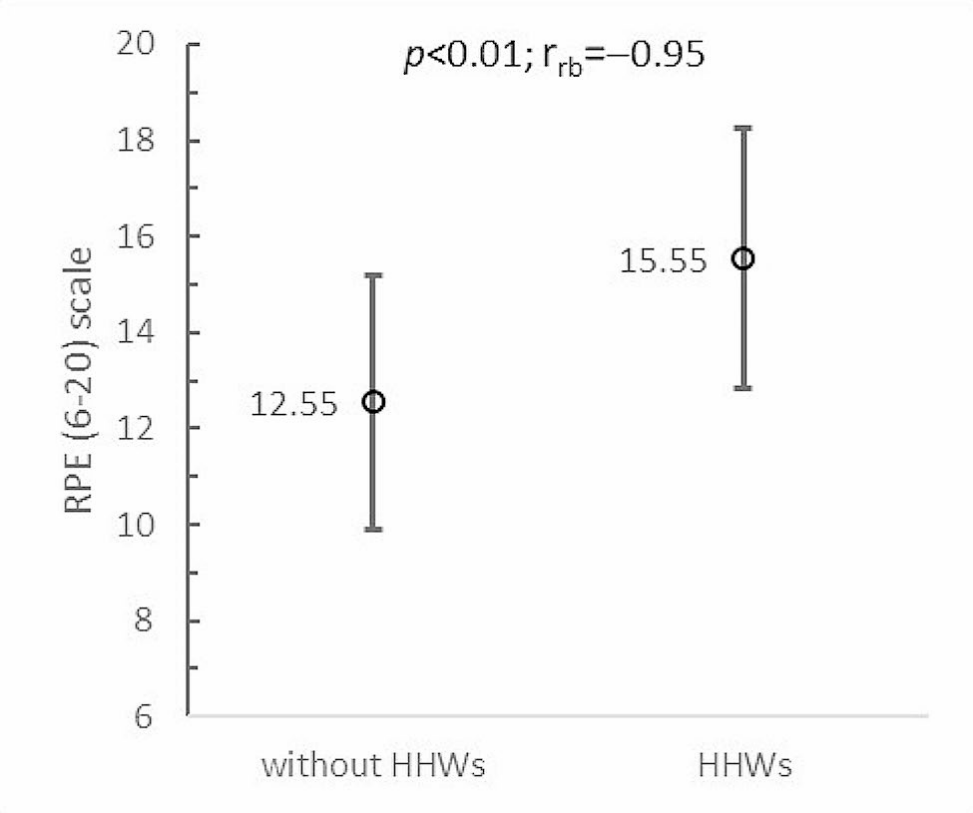
Rating of perceived exertion vs. upper limb loading: HHWs - hand-held weights; *p* - *p*-value; r_rb_ - rank-biserial correlation coefficient

Furthermore, statistically significant differences in results were found during the participants’ subjective assessment of the intensity of the physical exercise analyzed on the Borg’s RPE (6–20) scale. Boxing training in VR without HHWs was rated by paraboxers as significantly less intense than training with additional upper limb loading *(p = 0*.01; r_rb_=-0.95). In the former case, perceived exercise load was at 12.55 ± 2.64 points on Borg’s RPE (6–20) scale, while in the latter - at 15.55 ± 2.71 points (Fig. 6). Comparison of this assessment with the classification of PA intensity [49] shows that the wheelchair boxers rated PA as moderate for exercise without HHWs (which is similar to the objective measurement using heart rate monitor) and vigorous for exercise with HHWs. Spearman correlation analysis between subjective and objective measures of exercise intensity shows that there was a statistically significant *(p < 0*.03) relationship between intensity ratings estimated from %HRmax and Borg’s RPE (6–20) scale for PA in VR with HHWs (r_S_=0.64), whereas for exercise without HHWs, despite a relatively high correlation coefficient (r_S_=0.49), the relationship was statistically insignificant (*p* = 0.13).

### Wheelchair boxers’ satisfaction with PA in VR and their views on the usefulness of a virtual form of boxing training

**Fig. 7:**
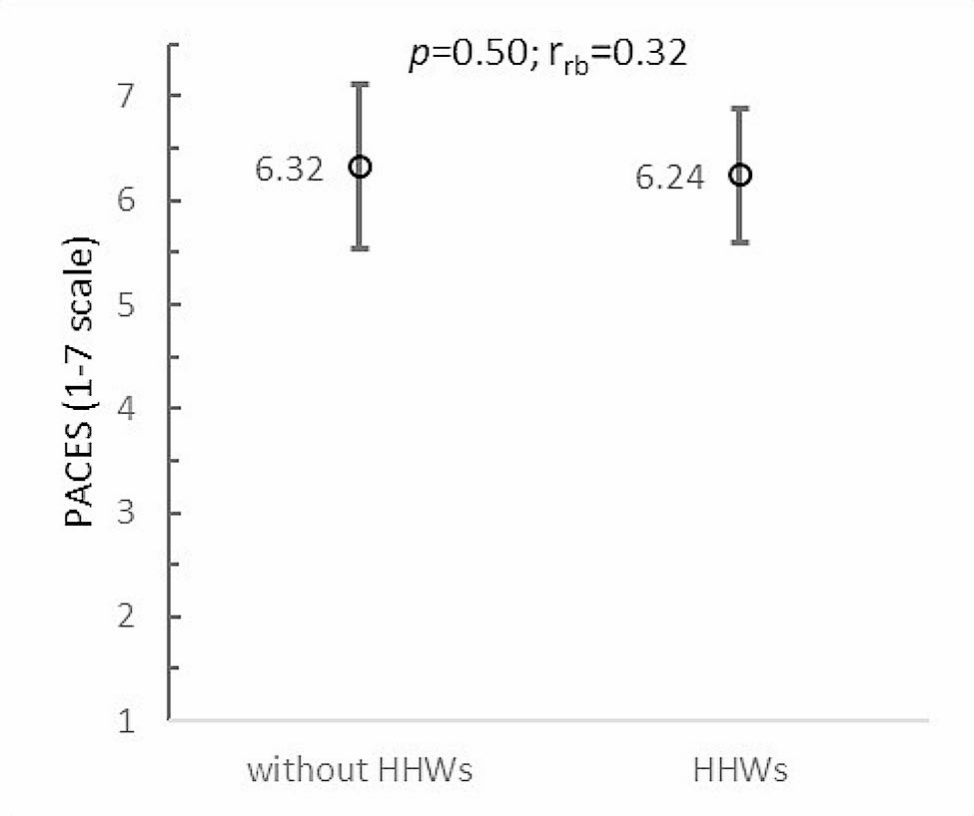
Satisfaction of wheelchair boxers with PA in VR vs. upper limb loading: HHWs - hand-held weights; *p* - *p*-value; r_rb_ - rank-biserial correlation coefficient; PACES - Physical Activity Enjoyment Scale

The PACES (1–7 scale) survey of wheelchair boxers found that athletes rated their satisfaction with boxing training in VR very highly both without HHWs (6.32 ± 0.79) and with HHWs (6.24 ± 0.64). The differences between these scores were small and statistically insignificant (*p* = 0.50; r_rb_=0.32) (Fig. 7).

## Discussion

The results of the study showed that the intensity of wheelchair boxing training in VR was at a moderate level. According to World Health Organization (WHO) guidelines, moderate-intensity efforts should be viewed as health-promoting PA [50]. Therefore, in terms of health benefits, the virtual environment exercises used in our study may not only complement the paraboxing training but also be offered as a health-promoting form of PA for other wheelchair users. A major problem for these people is movement and, consequently, the participation in organized forms of PA. VR technology, on the other hand, makes it possible to undertake various forms of PA in a small space, without having to leave home. Contrary to appearances, VR can be also used to socialize, which can be achieved using the multiplayer mode.

The health-promoting nature of exercise during immersive virtual reality video gaming (AVRGs) has also been documented by other authors [2–5, 10]. However, the studies refer to able-bodied users without fitness deficits. There is a lack of studies on the assessment of PA in physically disabled wheelchair users, i.e. people who use AVRGs in a seated position. It is a form of physical activity that is mainly limited to exercise performed with the upper limbs.

Interestingly, no significant effect of HHWs on the exercise intensity of paraboxers was found during boxing training in VR, especially as other studies show that the application of a small additional load on the upper limbs significantly increases the intensity of PA during exercise in VR [3]. The research was carried out using a popular AVRG (Beat Saber). The game involves the user using lightsabres to cut through virtual cubes moving toward him or her to the beat of music. The authors studied a group of young adults and showed that the additional loading of the wrists with 0.5 kg HHWs caused the relatively low intensity of exercise during playing Beat Saber to increase significantly to moderate. Studies of PA intensity in VR using Velcro-fastened weights were also carried out by another research group [4]. The authors assessed the effect of ankle weights (AW) weighing 2 kg on exercise intensity during AVRG involving the lower limbs. Participants used a multidirectional treadmill with leg loading and in non-AW conditions. The authors demonstrated that both forms of PA were characterized by high levels of PA intensity, but training with AW significantly increased exercise intensity in VR. The cited results of the study indicate that a small load to the upper limbs in the form of HHWs or on the lower limbs using AW during VR exercise in healthy individuals results in a significant increase in exercise intensity. In contrast, this pattern was not observed for paraboxers in our study. This may be because people after spinal cord injury (SCI) may have a reduced exercise heart rate [51]. This would be confirmed by the paraboxers’ subjective perceptions about PA during boxing training in VR. The athletes perceived exercise in VR without HHWs as moderate, while with HHWs, their assessment differed significantly, and the level of exercise load estimated by the participants on the Borg scale can be described as intense. This shows that the participant felt significantly more fatigued when training with HHWs than without HHWs, although objective measurements did not confirm this relationship.

Also interesting is the high correlation between PA intensity scores estimated from %HRmax and Borg’s RPE (6–20) scale for PA in VR with HHWs (r_S_=0.64), with a statistically insignificant correlation of scores for exercise without HHWs. It is worth mentioning that similar correlations were also observed in our previous studies, in which we found that without the application of additional load, the results of subjective and objective measurements of exercise intensity in VR did not correlate with each other, whereas after the application of additional resistance, the correlation was observed [3]. This fact is difficult to explain unequivocally, although it can be supposed that the additional small load applied during PA in VR makes the fatigue experienced by users more relevant to the actual fatigue than when physical exercise in VR is performed without load. This conclusion can be drawn from the analysis of studies by other authors, who have found that being in a virtual environment reduces the intensity of perception of various stimuli, e.g. pain, because VR, by stimulating different senses, distracts the immersed person from the problem [52–55]. The phenomenon is referred to as cognitive distraction. If it occurs during exercise in VR, it can alleviate the discomfort associated with hard training. Confirmation of this assumption could be of great importance in the case of individuals with disabilities, who are generally characterized by reduced levels of fitness and physical capacity. This would allow these people to perform more intense and longer exercises in a virtual environment. The few studies published to date suggest that VR may be useful in distracting from unpleasant bodily sensations occurring during aerobic PA in overweight and obese children [56] and in reducing negative sensations associated with the performance of isometric exercises [57].

The analysis of the wheelchair boxers’ satisfaction with PA in VR based on the PACES survey reveals that this form of training suits disabled athletes, as evidenced by the high rating given to it by the study participants. The attractiveness of PA in VR has also been assessed in previous studies, yielding similar results [3, 4, 8–12]. However, these studies concerned physically healthy people. There is a lack of this type of research on wheelchair users. Based on the analysis of the results of the PACES survey, it is worth noting that the paraboxers in our study rated their satisfaction with exercise in VR at similar levels both without upper limb loading and with HHWs, and the differences between these ratings were small and statistically insignificant. This shows that the additional load on the wrists in the form of 0.5 kg HHWs during exercise in VR did not disturb the paraboxers. Furthermore, with the question in the questionnaire “Did you experience any discomfort from the extra weight when playing with hand-held weights?“, no respondent answered in the affirmative. This may result from the cognitive distraction mentioned earlier. Similar conclusions in this respect have also been presented in previous studies [3]. The results of the evaluation of satisfaction with PA in VR of wheelchair paraboxers encourage further research explorations related to the experiences and perceptions of people with physical disabilities when exercising in a virtual environment to identify factors influencing the attractiveness of this form of exercise. The data collected in this way could help the developers of AVGs and virtual training and rehabilitation programs to guide further development. It is important to note that satisfaction is an important predictor of regular healthy PA and that how people feel during exercise determines their future involvement in training or therapies [58].

Finally, it should be emphasized that the paraboxers in our study only used one form of training in VR involving interaction with virtual objects, which is only a substitute for the possibilities offered by virtual technology. There are now boxing apps where users can interact with other fighters in a multiplayer mode and not only exercise together but also participate in a boxing bout. It is worth noting that for some paraboxers (e.g. those with ventriculoperitoneal shunts), this may be the only safe way to compete in this sport. With such applications, paraboxers can compete not only against each other but also against able-bodied people. VR thus becomes a ‘space’ in which it is possible for able-bodied people to compete with those with disabilities. It allows people with musculoskeletal dysfunctions to reject complexes and limitations and feel like able-bodied athletes. Even more realistic competition will be possible when haptic technology costumes and gloves are included with the app to increase the sensation of immersion and interaction. Haptic technology, which uses mechanical communication with users through the sense of touch [59], will allow users to feel the punches inflicted by an opponent. The use of VR and haptics in the context of PA for people with disabilities needs to be looked at more broadly, as these technologies are likely to find applications in training and competition in many other sports.

## Limitations

Finally, it is important to mention the limitations of the present study. Undoubtedly one of them is the relatively small group of respondents. The reasons were given when characterizing the participants in the study, pointing out that wheelchair boxing is a newly established sport and the population of paraboxers in Poland affiliated with clubs and associations is only a few dozen. It is therefore important to emphasize that in our study, the athletes, contrary to appearances, represented a relatively large percentage of wheelchair boxers in Poland. Another limitation is the method used to evaluate exercise intensity based on heart rate monitoring. A more precise method of evaluating exercise intensity, such as indirect calorimetry would be more appropriate. In our study, we decided to use heart rate monitoring because of the multifaceted nature of the measurements. In addition to the objective assessment of PA intensity, users’ subjective severity of perceived exertion, and its attractiveness were also assessed. Therefore, there was concern that the masks used in calorimetry might cause discomfort and hinder the subjective assessment of participants’ perceptions.

## Conclusion

The present study determined the exercise intensity in a group of paraboxers during a 15-minute boxing workout using the FitXR app, which, based on the results, was at a moderate level beneficial to health. The use of an additional load in the form of half-kilogram weights attached to the wrists does not significantly increase the physical intensity of paraboxers during virtual training. Paraboxers highly rated the attractiveness of PA during virtual boxing training. Therefore, it seems that the FitXR app may be a pleasant and beneficial complement to conventional boxing exercises for wheelchair boxers.

## Practical applications and implications for further research

Due to the great attractiveness of PA in VR and the dynamic development of immersive information technologies, virtual training may quickly become not only an important supplement to conventional forms of exercise, but even an alternative solution. Even more so because, as it turns out, the intensity of this type of exercise is relatively high, which means that it can potentially bring health benefits. Therefore, the exercises in the virtual environment used in our research may not only complement the training of paraboxers, but also be a proposal of a health-related form of PA for other disabled people in wheelchairs who have problems with free movement and using organized forms of PA. VR technology allows you to perform various attractive exercises in a small space, without having to leave your home. It is even possible to establish social contacts in a virtual environment thanks to the multiplayer mode. Therefore, disabled athletes can interact with other players, train with them and even compete. Confrontation with healthy people is also possible. VR allows people with musculoskeletal dysfunctions to reject complexes and limitations and feel like fully functional individuals. Even greater opportunities will appear when haptic costumes and gloves become more available, which will enhance the feeling of immersion and interaction and enable users to communicate through the sense of touch.

Our research results encourage conducting similar research experiments. In the future, the research should include people with various disabilities and evaluate newly created training applications and AVRGs in the context of the intensity of physical exercise, the possibility of improving physical fitness with them, their attractiveness and usefulness for people with special needs.

### Electronic supplementary material

Below is the link to the electronic supplementary material.

**Additional file 1**. Questionnaire– Wheelchair boxers’ views on boxing training in VR.

## Data Availability

The datasets during and/or analyzed during the current study available from the corresponding author on reasonable request.

## References

[1] Furht B, editor. Immersive virtual reality. Encyclopedia of Multimedia. Boston, MA: Springer US; 2008. pp. 345–6.

[2] Giakoni-Ramírez F, Godoy-Cumillaf A, Fuentes-Merino P, Farías-Valenzuela C, Duclos-Bastías D, Bruneau-Chávez J (2023). Intensity of a physical Exercise Programme executed through immersive virtual reality. Healthc Basel Switz.

[3] Polechoński J, Zwierzchowska A, Makioła Ł, Groffik D, Kostorz K (2022). Handheld weights as an effective and comfortable way to increase Exercise intensity of physical activity in virtual reality: empirical study. JMIR Serious Games.

[4] Polechoński J, Kostorz K, Polechoński P (2023). Using ankle weights as an effective way to increase the intensity of physical activity while playing immersive virtual reality games on an omnidirectional treadmill. Appl Sci.

[5] Sousa CV, Hwang J, Cabrera-Perez R, Fernandez A, Misawa A, Newhook K (2022). Active video games in fully immersive virtual reality elicit moderate-to-vigorous physical activity and improve cognitive performance in sedentary college students. J Sport Health Sci.

[6] Chan ZYS, MacPhail AJC, Au IPH, Zhang JH, Lam BMF, Ferber R (2019). Walking with head-mounted virtual and augmented reality devices: effects on position control and gait biomechanics. PLoS ONE.

[7] Wodarski P, Jurkojć J, Bieniek A, Chrzan M, Michnik R, Polechoński J et al. The Analysis of the Influence of Virtual Reality on Parameters of Gait on a Treadmill According to Adjusted and Non-adjusted Pace of the Visual Scenery. In: International Conference on Information Technologies in Biomedicine. Springer; 2019. pp. 543–53.

[8] Dębska M, Polechoński J, Mynarski A, Polechoński P (2019). Enjoyment and intensity of physical activity in immersive virtual reality performed on innovative training devices in Compliance with recommendations for Health. Int J Environ Res Public Health.

[9] Feodoroff B, Konstantinidis I, Froböse I (2019). Effects of full body exergaming in virtual reality on Cardiovascular and muscular Parameters: cross-sectional experiment. JMIR Serious Games.

[10] Gomez D, Browne JD, Almalouhi A, Abundex M, Hu J, Nason S (2022). Muscle activity during immersive virtual reality exergaming incorporating an adaptive Cable Resistance System. Int J Exerc Sci.

[11] Polechoński J, Szczechowicz B, Ryśnik J, Tomik R (2024). Recreational cycling provides greater satisfaction and flow in an immersive virtual environment than in real life. BMC Sports Sci Med Rehabil.

[12] Zeng N, Pope Z, Gao Z (2017). Acute Effect of virtual reality Exercise Bike games on College Students’ physiological and psychological outcomes. Cyberpsychology Behav Soc Netw.

[13] Langer A, Polechoński J, Polechoński P, Cholewa J (2023). Ruler Drop Method in virtual reality as an Accurate and Reliable Tool for evaluation of reaction time of mixed Martial Artists. Sustainability.

[14] Polechoński J, Langer A (2022). Assessment of the relevance and reliability of reaction time tests performed in immersive virtual reality by mixed Martial arts fighters. Sensors.

[15] Specht J, Stegmann B, Gross H, Krakow K (2023). Cognitive training with Head-mounted Display virtual reality in Neurorehabilitation: pilot randomized controlled trial. JMIR Serious Games.

[16] Zhu S, Sui Y, Shen Y, Zhu Y, Ali N, Guo C (2021). Effects of virtual reality intervention on cognition and motor function in older adults with mild cognitive impairment or dementia: a systematic review and Meta-analysis. Front Aging Neurosci.

[17] Bauer ACM, Andringa G (2020). The potential of immersive virtual reality for cognitive training in Elderly. Gerontology.

[18] Campo-Prieto P, Rodríguez-Fuentes G, Cancela-Carral JM (2021). Can Immersive virtual reality videogames help Parkinson’s Disease patients? A case study. Sensors.

[19] Gsangaya MR, Htwe O, Selvi Naicker A, Md Yusoff BAH, Mohammad N, Soh EZF (2023). Comparison between the effect of immersive virtual reality training versus conventional rehabilitation on limb loading and functional outcomes in patients after anterior cruciate ligament reconstruction: a prospective randomized controlled trial. Asia-Pac J Sports Med Arthrosc Rehabil Technol.

[20] Hollywood R-A, Poyade M, Paul L, Webster A (2022). Proof of Concept for the use of immersive virtual reality in Upper Limb Rehabilitation of multiple sclerosis patients. Adv Exp Med Biol.

[21] Lim DY, Hwang DM, Cho KH, Moon CW, Ahn SY (2020). A fully immersive virtual reality method for Upper Limb Rehabilitation in spinal cord Injury. Ann Rehabil Med.

[22] Lima Rebêlo F, de Souza Silva LF, Doná F, de Sales Barreto A (2021). Souza Siqueira Quintans J. Immersive virtual reality is effective in the rehabilitation of older adults with balance disorders: a randomized clinical trial. Exp Gerontol.

[23] Mitrousia V, Giotakos O (2016). Virtual reality therapy in anxiety disorders. Psychiatriki.

[24] Szczepańska-Gieracha J, Jóźwik S, Cieślik B, Mazurek J, Gajda R (2021). Immersive virtual reality therapy as a support for Cardiac Rehabilitation: a pilot randomized-controlled trial. Cyberpsychology Behav Soc Netw.

[25] Zhou Z, Li J, Wang H, Luan Z, Li Y, Peng X (2021). Upper limb rehabilitation system based on virtual reality for breast cancer patients: development and usability study. PLoS ONE.

[26] Hoffman HG, Boe DA, Rombokas E, Khadra C, LeMay S, Meyer WJ (2020). Virtual reality hand therapy: a new tool for nonopioid analgesia for acute procedural pain, hand rehabilitation, and VR embodiment therapy for phantom limb pain. J Hand Ther off J Am Soc Hand Ther.

[27] Wittkopf PG, Lloyd DM, Coe O, Yacoobali S, Billington J (2020). The effect of interactive virtual reality on pain perception: a systematic review of clinical studies. Disabil Rehabil.

[28] Czech O, Wrzeciono A, Batalík L, Szczepańska-Gieracha J, Malicka I, Rutkowski S (2022). Virtual reality intervention as a support method during wound care and rehabilitation after burns: a systematic review and meta-analysis. Complement Ther Med.

[29] Soltani M, Drever SA, Hoffman HG, Sharar SR, Wiechman SA, Jensen MP (2018). Virtual reality analgesia for burn joint flexibility: a randomized controlled trial. Rehabil Psychol.

[30] Won AS, Bailey J, Bailenson J, Tataru C, Yoon IA, Golianu B (2017). Immersive virtual reality for Pediatric Pain. Child Basel Switz.

[31] Carus EG, Albayrak N, Bildirici HM, Ozmen SG (2022). Immersive virtual reality on childbirth experience for women: a randomized controlled trial. BMC Pregnancy Childbirth.

[32] Devigne L, Babel M, Nouviale F, Narayanan VK, Pasteau F, Gallien P (2017). Design of an immersive simulator for assisted power wheelchair driving. IEEE Int Conf Rehabil Robot Proc.

[33] Genova C, Biffi E, Arlati S, Redaelli DF, Prini A, Malosio M (2022). A simulator for both manual and powered wheelchairs in immersive virtual reality CAVE. Virtual Real.

[34] Hernandez-Ossa KA, Montenegro-Couto EH, Longo B, Bissoli A, Sime MM, Lessa HM (2020). Simulation System of Electric-Powered Wheelchairs for Training purposes. Sensors.

[35] Yang Y-S, Koontz AM, Hsiao Y-H, Pan C-T, Chang J-J (2021). Assessment of Wheelchair Propulsion performance in an immersive virtual reality Simulator. Int J Environ Res Public Health.

[36] Petri K, Emmermacher P, Danneberg M, Masik S, Eckardt F, Weichelt S (2019). Training using virtual reality improves response behavior in karate kumite. Sports Eng.

[37] Qian J, McDonough DJ, Gao Z (2020). The effectiveness of virtual reality Exercise on Individual’s physiological, psychological and rehabilitative outcomes: a systematic review. Int J Environ Res Public Health.

[38] Todorov K, Manolova A, Chervendinev G. Immersion in Virtual Reality Video Games for Improving Physical Performance Measures: A Review. In: 2019 27th National Conference with International Participation (℡ECOM). 2019. pp. 35–8.

[39] Michalski SC, Szpak A, Saredakis D, Ross TJ, Billinghurst M, Loetscher T (2019). Getting your game on: using virtual reality to improve real table tennis skills. PLoS ONE.

[40] Oagaz H, Schoun B, Choi M-H (2022). Performance improvement and skill transfer in table tennis through training in virtual reality. IEEE Trans Vis Comput Graph.

[41] Rose FD, Attree EA, Brooks BM, Parslow DM, Penn PR, Ambihaipahan N (2000). Training in virtual environments: transfer to real world tasks and equivalence to real task training. Ergonomics.

[42] Archambault P, Routhier F, Gagnon D, Miller W (2018). Usability and efficacy of a virtual reality simulator for power wheelchair training. Ann Phys Rehabil Med.

[43] Vailland G, Gaffary Y, Devigne L, Gouranton V, Arnaldi B, Babel M. Vestibular Feedback on a Virtual Reality Wheelchair Driving Simulator: A Pilot Study. In: 2020 15th ACM/IEEE International Conference on Human-Robot Interaction (HRI). 2020. pp. 171–9.

[44] Tanaka H, Monahan KD, Seals DR (2001). Age-predicted maximal heart rate revisited. J Am Coll Cardiol.

[45] Riebe D, Ehrman JK, Liguori G, Magal M, Medicine. AC of S. ACSM’s guidelines for exercise testing and prescription. Wolters Kluwer; 2018.

[46] Borg G (1970). Perceived exertion as an indicator of somatic stress. Scand J Rehabil Med.

[47] Scherr J, Wolfarth B, Christle JW, Pressler A, Wagenpfeil S, Halle M (2013). Associations between Borg’s rating of perceived exertion and physiological measures of exercise intensity. Eur J Appl Physiol.

[48] Mullen SP, Olson EA, Phillips SM, Szabo AN, Wójcicki TR, Mailey EL (2011). Measuring enjoyment of physical activity in older adults: invariance of the physical activity enjoyment scale (paces) across groups and time. Int J Behav Nutr Phys Act.

[49] Piepoli MF, Hoes AW, Agewall S, Albus C, Brotons C, Catapano AL (2016). 2016 European guidelines on cardiovascular disease prevention in clinical practice. Eur Heart J.

[50] WHO. WHO guidelines on physical activity and sedentary behaviour: web annex: evidence profiles. 2020. 33369898

[51] Jacobs PL, Nash MS (2004). Exercise recommendations for individuals with spinal cord injury. Sports Med Auckl NZ.

[52] Hoffman HG, Chambers GT, Meyer WJ, Arceneaux LL, Russell WJ, Seibel EJ (2011). Virtual reality as an adjunctive non-pharmacologic analgesic for acute burn pain during medical procedures. Ann Behav Med.

[53] Jin W, Choo A, Gromala D, Shaw C, Squire P. A Virtual Reality Game for Chronic Pain Management: A Randomized, Controlled Clinical Study. In: MMVR. 2016. pp. 154–60. 27046570

[54] Jones T, Moore T, Choo J (2016). The impact of virtual reality on chronic pain. PLoS ONE.

[55] Tashjian VC, Mosadeghi S, Howard AR, Lopez M, Dupuy T, Reid M et al. Virtual reality for management of pain in hospitalized patients: results of a controlled trial. JMIR Ment Health. 2017;4. 10.2196/mental.7387 PMC5390112 28356241

[56] Baños RM, Escobar P, Cebolla A, Guixeres J, Alvarez Pitti J, Lisón JF (2016). Using virtual reality to distract overweight children from bodily sensations during Exercise. Cyberpsychology Behav Soc Netw.

[57] Matsangidou M, Ang CS, Mauger AR, Intarasirisawat J, Otkhmezuri B, Avraamides MN (2019). Is your virtual self as sensational as your real? Virtual reality: the effect of body consciousness on the experience of exercise sensations. Psychol Sport Exerc.

[58] Williams DM, Dunsiger S, Ciccolo JT, Lewis BA, Albrecht AE, Marcus BH (2008). Acute affective response to a moderate-intensity Exercise stimulus predicts physical activity participation 6 and 12 months later. Psychol Sport Exerc.

[59] Biswas S, Visell Y (2021). Haptic perception, mechanics, and Material technologies for virtual reality. Adv Funct Mater.

