# Supplementary material for: Application of immersive virtual reality in the training of wheelchair boxers: evaluation of exercise intensity and users experience additional load– a pilot exploratory study
Source: BMC Sports Sci Med Rehabil. 2024 Apr 10;16:80. doi: 10.1186/s13102-024-00878-6 (PMC11008040; doi:10.1186/s13102-024-00878-6)
Supplement: Supplementary file 1 — Additional file 1. Questionnaire– Wheelchair boxers’ views on boxing training in VR. [file 13102_2024_878_MOESM1_ESM.docx]

Questionnaire – Wheelchair boxers' views on boxing training in VR

*(Check one answer after each question)*

1. Did you experience any discomfort from the extra weight when playing with hand held weights?

Strongly disagree □, Disagree □, Somewhat disagree □, Neither agree nor disagree □,

Somewhat agree □, Agree □, Strongly agree □

1. With the right hardware and software, would you practice wheelchair boxing in VR?

Strongly disagree □, Disagree □, Somewhat disagree □, Neither agree nor disagree □,

Somewhat agree □, Agree □, Strongly agree □

1. Would you recommend other athletes to practice wheelchair boxing in VR?

Strongly disagree □, Disagree □, Somewhat disagree □, Neither agree nor disagree □,

Somewhat agree □, Agree □, Strongly agree □

1. Do you think that practicing wheelchair boxing in VR is more enjoyable than

performing conventional boxing exercises?

Strongly disagree □, Disagree □, Somewhat disagree □, Neither agree nor disagree □,

Somewhat agree □, Agree □, Strongly agree □

1. Do you think practicing boxing in VR could complement conventional wheelchair boxing training?

Strongly disagree □, Disagree □, Somewhat disagree □, Neither agree nor disagree □,

Somewhat agree □, Agree □, Strongly agree □

1. In your opinion, can boxing training in VR benefit wheelchair boxers?

Strongly disagree □, Disagree □, Somewhat disagree □, Neither agree nor disagree □,

Somewhat agree □, Agree □, Strongly agree □
